# bpMRI and mpMRI for detecting prostate cancer: A retrospective cohort study

**DOI:** 10.3389/fsurg.2022.1096387

**Published:** 2023-01-16

**Authors:** Yongsheng Pan, Cheng Shen, Xinfeng Chen, Dongliang Cao, Jie Jiang, Wei Xu, Chen Ji, Xiaodong Pan, Bing Zheng

**Affiliations:** ^1^ Department of Urology, The Second Affiliated Hospital of Nantong University, Nantong, China; ^2^ Medical Research Center, The Second Affiliated Hospital of Nantong University, Nantong, China

**Keywords:** biparametric magnetic resonance imaging, multiparameter magnetic resonance imaging, prostate cancer, clinically significant prostate cancer, prostate specific antigen

## Abstract

**Background:**

We aimed to compare the detection rates of prostate cancer (PCa) and clinically significant prostate cancer(csPCa) by biparametric (bp-) and multiparameter magnetic resonance imaging (mpMRI).

**Materials and Methods:**

A total of 699 patients who underwent transperineal prostate biopsy in the Department of Urology, the Second Affiliated Hospital of Nantong University from January 2018 to December 2021 were retrospectively reviewed. Multivariate analysis was used to explore the influencing factors associated with the detection rates of PCa and csPCa. According to MRI examination before biopsy, the patients were divided into bpMRI group and mpMRI group. The detection rates of PCa and csPCa by bpMRI and mpMRI were compared. Furthermore, stratified analysis was performed for patients in these two groups to compare the detection rates of PCa and csPCa at different tPSA intervals, different prostate volume (PV) intervals and different PI-RADS V2 scores.

**Results:**

A total of 571 patients were finally analyzed in this study after exclusion, and the overall detection rate of PCa was 54.5%. Multivariate analysis showed that patient age, tPSA level, prostate volume and PI-RADS V2 score were independent risk factors affecting the detection rates of PCa and csPCa. The detection rates of PCa and csPCa by bpMRI and mpMRI were comparable (51.3% vs. 57.9%, 44.0% vs. 48.0%, both *P* > 0.05), with no statistical significance. In the tPSA 10–20 ng/ml interval, the detection rates of PCa (59.72% vs. 40.35%, *P* = 0.011) and csPCa (51.39% vs. 28.82%, *P* = 0.005) by mpMRI were significantly higher than those by bpMRI, while in other tPSA interval (tPSA < 4 ng/ml, 4–10 ng/ml, 20–100 ng/ml), different PVs (≤30 ml, 30–60 ml, >60 ml) and different PI-RADS V2 scores (3, 4, and 5), the detection rates of PCa and csPCa were comparable between the two groups.

**Conclusion:**

For detecting PCa and csPCa, bpMRI and mpMRI had similar diagnostic efficacies, whereas mpMRI detected more PCa and csPCa in the tPSA interval of 10–20 ng/ml.

## Introduction

Prostate cancer (PCa) is the second most common male malignant cancer worldwide, and its death rate ranks sixth (1). Nowadays, multiparameter magnetic resonance imaging (mpMRI) plays an important role in the detection of prostate cancer. Due to the application of mpMRI, the detection rates of PCa and clinically significant prostate cancer (csPCa) has significantly improved in the past decade (2–4).

The latest Prostate Image Reporting and Data System (PI-RADS) proposed that the DCE sequence (dynamic contrast enhancement) in mpMRI was with limited efficacy for diagnosing PCa sometimes (2). Only when the suspicious lesion is located in the peripheral zone of the prostate with a PI-RADS score of 3–4 in the T2WI sequence may it help increase the detection rate of csPCa. In clinical practice, some physicians only use the DCE sequence as an “insurance” sequence when the DWI sequence (diffusion weighted imaging) was not enough to make a definitive diagnosis of prostate cancer due to human factors or insufficient signal-to-noise ratio. In recent years, a number of studies have shown the positive effect of biparametric magnetic resonance imaging (bpMRI) on improving the detection rate of csPCa (5, 6). Though suggestions by the European Society of Urogenital Radiology to use complete multiparametric (mp) T2-weighted/diffusion weighted imaging(DWI)/dynamic contrast enhancement (DCE) acquisition for all prostate MRI examinations, the real advantage of functional DCE remains a matter of debate (7). Therefore, the PI-RADS Steering Committee supported the ongoing study of bpMRI in various clinical protocols and recognized the potential advantages of bpMRI, including the avoidance of contrast-related adverse reactions, shorter test times and lower costs (2).

In the present study, we analyzed the clinical data of 699 patients who underwent transperineal prostate biopsy in our center. The detection rates of PCa and csPCa by bpMRI and mpMRI were compared at different tPSA intervals, different prostate volumes and different PI-RADS V2 scores.

## Materials and methods

### Study design and study population

This is a retrospective study approved by the institutional review board and written informed consent was obtained from all patients. From January 2018 to December 2021, a total of 699 patients suspicious of prostate cancer (PSA ≥ 4 ng/ml, or abnormal digital rectal examination results, or abnormal ultrasound or MRI examination results) underwent transperineal prostate biopsy in our hospital. Patients with a previous prostate biopsy history or a prior diagnosis of prostate cancer were excluded.

### Surgical method

In this study, all patients underwent transperineal prostate biopsy. They were placed in the lithotomy position, routinely disinfected, and draped with a sterile hole towel. Then 1% lidocaine was used for subcutaneous local infiltration anesthesia of the puncture site in the perineal region. The rectal ultrasound probe was placed in the rectum, and infiltration anesthesia deep to the extraprostatic capsule was done at the puncture site under direct ultrasound guidance. After that, combined cognitive MRI-targeted biopsy and systematic biopsy were performed using 18G puncture needle (model: MC1820, Bard Peripheral Vascular). Cognitive MRI-targeted biopsy was performed with 2 cores per targeted lesion, followed by 12-core systematic biopsy.

### The outcomes

The primary outcome was to evaluate the detection rates of PCa and csPCa by bpMRI and mpMRI based on pathological results of prostate biopsies. The secondary outcome was to analyze the detection rates of PCa and csPCa by bpMRI and mpMRI stratified by tPSA level, PV (prostate volume), and PI-RADS score. In different tPSA intervals (<4 ng/ml, 4–10 ng/ml, 10–20 ng/ml, and 20–100 ng/ml), the detection rates of PCa and csPCa by bpMRI and mpMRI were compared. Prostate volume was calculated according to magnetic resonance imaging measurements (V = anteroposterior diameter * transverse diameter * longitudinal diameter * 0.52), and the detection rates of PCa and csPCa by bpMRI and mpMRI were compared in different PV intervals (≤ 30 ml, 30–60 ml, and >60 ml). Also, the detection rates of PCa and csPCa by bpMRI and mpMRI were compared stratified by PI-RADS V2 scores (3, 4, and 5).

### Histopathological evaluation and tumor significance

All biopsy samples were reviewed by the same genitourinary pathologist (>15 years of experience). For each prostate cancer-positive biopsy core, the location, Gleason score (GS) based on the International Society of Urological Pathology 2005 consensus (8), and percentage of cancerous tissue per core were determined. In addition, patients were allocated using the International Society of Urological Pathology 2014 consensus Gleason-grade groups (9) based on the GS scoring criteria (8). In this study, csPCa was defined as ≥ Gleason score of 3 + 4 = 7.

### Statistical analysis

In this study, SPSS 23.0 (IBM) software was used for statistical analysis, and patient characteristics were reported using descriptive statistical methods. Continuous variables such as age, PSA level, PSA density, and prostate volume were compared using the t-test. All continuous variables were expressed in the form of mean ± standard deviation, and the chi-square test was applied for categorical variables, *P* < 0.05 was considered statistically significant.

### The results

**Figure 1:**
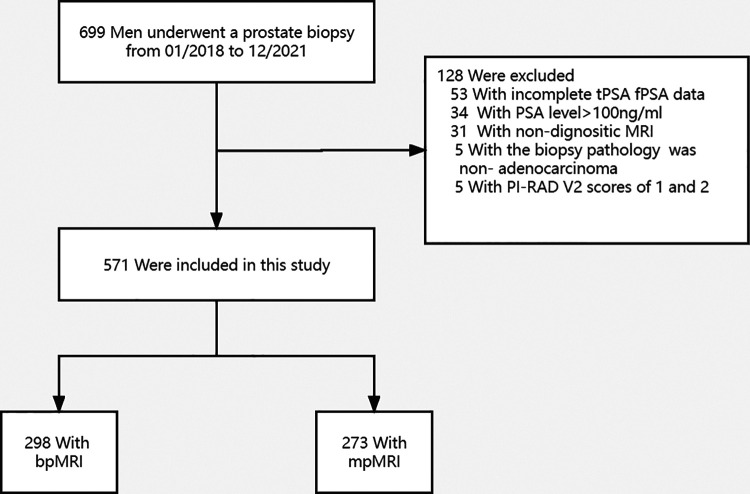
Flowchart of the study population. mpMRI, multiparametric magnetic resonance imaging; bpMRI, Biparametric magnetic resonance imaging; tPSA, Total prostate-specific antigen; fPSA, Free prostate-specific antigen; PI-RADS V2, Prostate Imaging Report Data System, version 2.

Between January 2018 and December 2021, a total of 699 patients underwent transperineal prostate biopsies in our hospital. Of these 699 patients, 128 were excluded for various reasons (Figure 1), such as 53 patients without complete tPSA and fPSA values, 34 patients with tPSA level greater than 100 ng/ml, 31 patients without MRI examination, 5 patients with PI-RADS V2 of 1 or 2, and 5 patients with biopsy pathological results of non-adenocarcinoma type. The remaining 571 patients met the study inclusion criteria and were available for the final analysis. The baseline characteristics of the patients are provided in Supplementary Table S1, and statistical tests revealed that the bpMRI group and mpMRI group did not have a significant difference regarding age, tPSA levels, PV, PSA density (PSAD), and PI-RADS V2 score (all *P *> 0.05). The overall detection rates of PCa and csPCa were comparable between the bpMRI group and mpMRI group (51.3% vs. 57.9%, 44.0% vs. 48.0%, both *P* > 0.05), with no statistical significance.

The results of multivariate analysis showed that patients' age, tPSA level, PV, and PI-RADS V2 score were independent risk factors for PCa and csPCa detection (all *P *< 0.05), regardless of MRI patterns (*P* > 0.05) (Supplementary Tables S2, S3). Based on the results of multivariate analysis, we chosed three independent risk factors responsible for the detection of PCa and csPCa, tPSA level, PV, and PI-RADS V2 score, for further analysis. According to tPSA levels, patients were divided into four subgroups tPSA < 4 ng/ml, 4 ≤ tPSA ≤ 10 ng/ml, 10 < tPSA ≤ 20 ng/ml, and 20 < tPSA ≤ 100 ng/ml. The results (Supplementary Table S4) showed that in the tPSA 10–20 ng/ml interval, the detection rates of PCa (59.72% vs. 40.35%, *P* = 0.0109) and csPCa (51.39% vs. 28.82%, *P* = 0.0129) by mpMRI were significantly higher than those by bpMRI, while in other tPSA intervals (tPSA < 4 ng/ml, 4–10 ng/ml, 20–100 ng/ml), the detection rates of PCa and csPCa by bpMRI and mpMRI were comparable (all *P *> 0.05), with no statistical significance (all *P *> 0.05).

In this study, prostate volumes were calculated based on MRI measurements. In order to compare the detection rates of PCa and csPCa by bpMRI and mpMRI in different prostate volume intervals, patients were divided into three subgroups, PV ≤ 30 ml, 30–60 ml, and >60 ml. However, the results (Supplementary Table S5) showed that the detection rates of PCa and csPCa are comparable between the bpMRI and mpMRI group in different prostate volume intervals (all *P *> 0.05), with no statistical significance. In addition, we also found that the detection rates of PCa and csPCa by bpMRI and mpMRI were comparable at different PI-RADS V2 scores (3, 4, and 5) (all *P *> 0.05), with no statistical significance (Supplementary Table S6).

## Discussion

In recent years, MRI-fusion biopsy has been widely used for diagnosis of prostate cancer in clinical practice, improving the detection rate of clinically significant prostate cancer (10). With the increasing demand for MRI of the prostate, doubts have been raised about whether a comprehensive examination can be obtained while saving time and cost. We all know that the use of dynamic contrast-enhanced imaging (DCE) requires intravenous contrast, which prolongs the time of MRI examination, increases the cost burden for patients, and may even cause contrast-related adverse effects. Alternatively, the examination can be completed in less than 15 min utilizing a bpMRI pattern, making imaging non-traumatic (11, 12). At present, more and more studies have evaluated the diagnostic efficacies of bpMRI and mpMRI methods, and many authors emphasize that the diagnostic efficiency of the two regimens is overlapping (13–15). The results of a multicenter multi-reader trial (PROMIS) showed no significant difference between bpMRI and mpMRI in excluding csPCa (16). As stated in the PIRADS Committee position paper, MRI quality is critical in the bp approach because image quality is sufficient to detect or exclude csPCa (17, 18).

In the present study, we analyzed the detection rates of PCa and csPCa in 571 men who underwent bpMRI or mpMRI, and found that the detection rates of PCa and csPCa by the two MRI modalities were comparable and with no statistical significance. These results suggest that bpMRI can also be used as one of the auxiliary diagnostic modality for prostate cancer, and the diagnostic efficiency of bpMRI for PCa and csPCa is not inferior to that of mpMRI.

As recommended by the PIRADS committee, the current role of DCE is limited to type 3 lesions to determine the nature of equivocal lesions (7). Although the sensitivity of DCE is high, but its specificity may be low. High sensitivity is true positive because it means the proportion of positives correctly identified, while specificity is true negative, which means positive results have the possibility of false positives and additional biopsies may be required. Some investigators have found that mpMRI-based diagnostic modality for prostate cancer may lead to more false-positive results (12, 19). In clinical practice, reducing false positive results of MRI means decreasing prostate biopsies in patients, which can reduce the biopsy-related complications such as pain, bleeding, infection, etc. and avoid overdiagnosis and overtreatment. In addition, Kuhl et al. found no significant difference in the diagnostic accuracy of bpMRI and mpMRI in repeated biopsies of 542 men with elevated PSA values (20, 21), which further suggested that the use of bpMRI with diagnostic specificity as an auxiliary modality for prostate cancer may be able to decrease unnecessary prostate biopsies.

Since the diagnostic performance of the bpMRI method is not inferior to that of the mpMRI, the application of the bpMRI method requires high image quality and reader expertise (7). In this study, we retrospectively analyzed the clinical data of 699 patients who underwent prostate biopsy from January 2018 to March 2021. The effect of baseline data of patients on the positive rate of biopsy was analyzed. According to the MRI examination before biopsy, the patients were divided into bpMRI group and mpMRI group, and baseline characteristics between the two groups were comparable. Stratified analysis was performed for patients in the two groups according to tPSA levels, PVs and PI-RADS V2 scores to compare the detection rates of PCa and csPCa by bpMRI and mpMRI. The results of stratified analysis showed that in the tPSA 10–20 ng/ml interval, the detection rate of PCa (58.1% vs. 31.7%, *P* = 0.004) and csPCa (46.8% vs. 20.6%, *P* = 0.002) by mpMRI were significantly higher than those by bpMRI; in the other tPSA intervals, the detection rates of the two MRI modalities were comparable, with no statistical significance. Our study indicates that when patients' tPSA values are in the 10–20 ng/ml interval, they should undergo mpMRI examination which may improve the detection rates of PCa and csPCa. While in other tPSA intervals, they can only undergo bpMRI examinations for detecting PCa and csPCa instead of mpMRI examinations. We speculate that this difference may be due to the fact that prostate cancer lesions do not perform significantly on bpMRI images in the tPSA 10–20 ng/ml interval, while the addition of DCE sequences can improve the sensitivity of interpretation of suspicious lesions. However, it needs to be further studied.

As we all know, the tPSA 4–10 ng/ml is a gray area for prostate cancer determination. When patients' tPSA values is in the gray area, it is often necessary to refer to fPSA and other PSA-related derived indicators such as f/tPSA, PSAD and PSAV (PSA rate) [20]. We supposed mpMRI had higher diagnostic efficiency of PCa and csPCa in the tPSA gray area compared to bpMRI, while further analysis revealed that mpMRI was not superior to bpMRI in this tPSA interval. In addition, in other tPSA intervals (tPSA < 4 ng/ml, 20–100 ng/ml), different PVs (≤30 ml, 30–60 ml, >60 ml), and different PI-RADS V2 scores (3, 4, and 5), the detection rates of PCa and csPCa were comparable between the two groups, and the difference was not statistically significant.

## Limitations

Our study has some limitations (1). This was a single-center retrospective study with a relatively small number of patients, and the current results should be validated in a prospective multicenter clinical trial (2). In this study, patients were divided into the bpMRI group and mpMRI group according to MRI modalities before biopsy. However, the image interpretation of bpMRI and mpMRI was not performed for the same patients undergoing mpMRI examination, respectively. In further studies, the image interpretation of bpMRI and mpMRI could be performed for the same patient to compare the detection rates of PCa and csPCa between the two MRI modalities.

Despite these limitations, our findings validate bpMRI as an alternative to mpMRI for detecting PCa and csPCa in clinical practice. Besides, as a more rapid and simple modality, bpMRI is feasible in our center.

## Conclusion

The overall detection rates of PCa and csPCa by bpMRI and mpMRI were comparable, but mpMRI detected more PCa and csPCa in the tPSA interval of 10–20 ng/ml. In other tPSA intervals, bpMRI could be an alternative to mpMRI for detecting PCa and csPCa, regardless of different prostate volumes and PI-RADS scores.

## Data Availability

The original contributions presented in the study are included in the article/**Supplementary Material**, further inquiries can be directed to the corresponding author/s.
