# Supplementary material for: bpMRI and mpMRI for detecting prostate cancer: A retrospective cohort study
Source: Front Surg. 2023 Jan 16;9:1096387. doi: 10.3389/fsurg.2022.1096387 (PMC9885087; doi:10.3389/fsurg.2022.1096387)
Supplement: Supplementary file 1 [file Table1.doc]

**Table 1: Basic clinical information of bpMRI group and mpMRI group**

| Characteristic | bpMRI  (n=298) | mpMRI  (n=273) | *P* |
| --- | --- | --- | --- |
| Age, median(IQR), yr | 70.97±7.95 | 71.14±8.34 | 0.796 |
| tPSA（ng/ml） | 21.7±21.02 | 20.16±19.28 | 0.363 |
| PV（ml） | 47.81±28.96 | 45.02±27.1 | 0.237 |
| PSAD（ng/ml^2^） | 0.61±0.76 | 0.56±0.64 | 0.384 |
| PI-RADS V2 |  |  | 0.301 |
| 3 | 96（32.2%） | 72（26.4%） |  |
| 4 | 115（38.6%） | 112（41.0%） |  |
| 5 | 87（29.2%） | 89（32.6%） |  |
| PCa | 153（51.3%） | 158（57.9%） | 0.130 |
| csPCa | 131（44.0%） | 131（48.0%） | 0.356 |

Abbreviations: bpMRI, two-parameter magnetic resonance; mpMRI, multi-parameter magnetic resonance; tPSA, Total prostate specific antigen; PV, prostate volume; PSAD, PSA density; PI-RADS V2, Prostate Imaging Report Data System, version 2; PCa, prostate cancer; CsPCa, clinically significant prostate cancer.

**Table 2 :Multivariate logistics regression analysis to identify independent contributors to PCa detection**

| Independent variable | *β* | SE | Wald | *p* | OR | 95%CI |
| --- | --- | --- | --- | --- | --- | --- |
| Age, yr | 0.091 | 0.016 | 31.355 | <0.001 | 1.095 | 1.061-1.130 |
| Tpsa, ng/mL | 0.034 | 0.009 | 15.192 | <0.001 | 1.034 | 1.017-1.052 |
| PV | -0.047 | 0.006 | 58.680 | <0.001 | 0.954 | 0.943-0.966 |
| MRI |  |  |  |  |  |  |
| bpMRI |  |  |  |  | reference |  |
| mpMRI | 0.240 | 0.235 | 1.046 | 0.306 | 1.272 | 0.802-2.015 |
| PI-RADS-V2 |  |  |  |  |  |  |
| 3 |  |  |  |  | reference |  |
| 4 | 1.380 | 0.269 | 26.393 | <0.001 | 3.976 | 2.348-6.732 |
| 5 | 3.183 | 0.387 | 67.539 | <0.001 | 24.112 | 11.287-51.510 |

Abbreviations: bpMRI, two-parameter magnetic resonance; mpMRI, multi-parameter magnetic resonance; tPSA, Total prostate-specific antigen; PV, prostate volume; PSAD, PSA density; PI-RADS V2, Prostate Imaging Report Data System, version 2; PCa, prostate cancer; csPCa, clinically significant prostate cancer; OR, odds ratio; CI, confidence interval.

**Table 3:Multivariate logistics regression analysis to identify independent contributors to csPCa detection**

| Independent variable | *β* | SE | Wald | *p* | OR | 95%CI |
| --- | --- | --- | --- | --- | --- | --- |
| Age | 0.082 | 0.016 | 26.316 | <0.001 | 1.086 | 1.052-1.121 |
| tPSA | 0.038 | 0.008 | 21.959 | <0.001 | 1.039 | 1.022-1.056 |
| PV | -0.041 | 0.006 | 43.010 | <0.001 | 0.960 | 0.948-0.971 |
| MRI |  |  |  |  |  |  |
| bpMRI |  |  |  |  | reference |  |
| mpMRI | 0.068 | 0.237 | 0.083 | 0.773 | 1.071 | 0.673-1.703 |
| PI-RADS-V2 |  |  |  |  |  |  |
| 3 |  |  |  |  | reference |  |
| 4 | 1.709 | 0.325 | 27.713 | <0.001 | 5.522 | 2.923-10.434 |
| 5 | 3.362 | 0.383 | 76.866 | <0.001 | 28.839 | 13.602-61.146 |

Abbreviations: MRI, magnetic resonance; bpMRI, two-parameter magnetic resonance; mpMRI, multi-parameter magnetic resonance; tPSA, Total prostate-specific antigen; PV, prostate volume; PI-RADS V2, Prostate Imaging Report Data System, version 2; csPCa, clinically significant prostate cancer; OR, odds ratio; CI, confidence interval;

**Table 4: Comparison of the detection rates of PCa and csPCa between bpMRI and mpMRI according to different tPSA intervals**

|  | bpMRI | mpMRI | *P* |
| --- | --- | --- | --- |
| tPSA<4ng/ml | n=7 | n=7 |  |
| PCa | 5（71.4%） | 6（85.7%） | 1.000 |
| csPCa | 4（57.1%） | 4（57.1%） | 1.000 |
| 4≤tPSA≤10ng/ml | n=86 | n=104 |  |
| PCa | 32（37.2%） | 41（39.4%） | 0.767 |
| csPCa | 25（29.1%） | 27（26.0%） | 0.744 |
| 10<tPSA≤20ng/ml | n=114 | n=72 |  |
| PCa | 46（40.4%） | 43（59.7%） | 0.011 |
| csPCa | 34（29.8%） | 37（51.4%） | 0.005 |
| 20<tPSA≤100ng/ml | n=91 | n=90 |  |
| PCa | 70（76.9%） | 68（75.6%） | 0.863 |
| csPCa | 68（74.7%） | 63（70.0%） | 0.509 |

Abbreviations: tPSA = total prostate-specific antigen; csPCa, clinically significant PCa; PCa, prostate cancer;

**Table 5: Comparison of the detection rates of PCa and csPCa between bpMRI and mpMRI according to different prostate volumes**

|  | bpMRI | mpMRI | *P* |
| --- | --- | --- | --- |
| PV≤30ml | n=85 | n=85 |  |
| PCa | 62（72.9%） | 67（78.8%） | 0.474 |
| csPCa | 55（64.7%） | 55（64.7%） | 1.000 |
| 30<PV≤60ml | n=144 | n=137 |  |
| PCa | 80（55.6%） | 77（56.2%） | 1.000 |
| csPCa | 66（45.8%） | 65（47.4%） | 0.812 |
| PV>60ml | n=69 | n=51 |  |
| PCa | 11（15.9%） | 14（27.5%） | 0.172 |
| csPCa | 10（14.5%） | 11（21.6%） | 0.340 |

Abbreviations: PV, prostate volume; csPCa, clinically significant PCa; PCa, prostate cancer;

**Table 6: Comparison of the detection rates of PCa and csPCa between bpMRI and mpMRI according to PI‐RADS V2 classification**

|  | bpMRI | mpMRI | *P* |
| --- | --- | --- | --- |
| PI-RADS V2 3 | n=96 | n=72 |  |
| PCa | 16（16.7%） | 14（19.4%） | 0.687 |
| csPCa | 7（7.3%） | 8（11.1%） | 0.423 |
| PI-RADS V2 4 | n=115 | n=112 |  |
| PCa | 59（51.3%） | 62（55.4%） | 0.595 |
| csPCa | 48（41.7%） | 48（42.9%） | 0.894 |
| PI-RADS V2 5 | n=87 | n=89 |  |
| PCa | 78（89.7%） | 82（92.1%） | 0.609 |
| csPCa | 76（87.4%） | 75（84.3%） | 0.667 |

Abbreviations: PI‐RADS V2, prostate imaging–reporting and data system version 2; csPCa, clinically significant PCa; PCa, prostate cancer;
